# Materials and Methods for All-Ceramic Dental Restorations Using Computer-Aided Design (CAD) and Computer-Aided Manufacturing (CAM) Technologies—A Brief Review

**DOI:** 10.3390/dj12030047

**Published:** 2024-02-22

**Authors:** Nestor Washington Solís Pinargote, Oleg Yanushevich, Natella Krikheli, Anton Smirnov, Sergey Savilkin, Sergey N. Grigoriev, Pavel Peretyagin

**Affiliations:** 1 Federal State Budgetary Educational Institution of the Higher Education Moscow State University of Technology “STANKIN”, 127055 Moscow, Russia; a.smirnov@stankin.ru (A.S.); s.grigoriev@stankin.ru (S.N.G.);; 2 Federal State Budgetary Educational Institution of the Higher Education “A.I. Yevdokimov Moscow State University of Medicine and Dentistry” of the Ministry of Healthcare of the Russian Federation, 127473 Moscow, Russia

**Keywords:** zirconia, zirconium dioxide, lithium disilicate, all-ceramic restoration, implants, crowns

## Abstract

The materials used in dentistry for the fabrication of all-ceramic restorations have undergone great and rapid developments over the last two decades. Among the most common ceramic materials in dentistry are those based on zirconium and lithium disilicate. Due to the properties of these materials, they are in great demand in the field of dental restoration production. Thus, dental restorations that will use those materials are commonly machined in CAD/CAM systems, which offer the possibility of manufacturing all-ceramic dental restorations in a very short period of time. This article reviews the modern materials in the field of all-ceramic dental restorations, their manufacturing processes, as well as what determines which ceramic materials are used for the production of CAD/CAM blanks and their production technology.

## 1. Introduction

**Figure 1:**
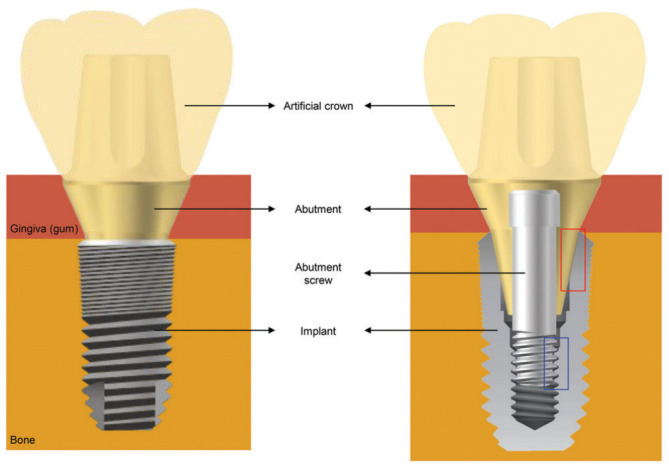
Basic structure of a dental implant system. Reproduced from [2] (CC BY-NC 3.0), published by The Royal Society of Chemistry’s, 2022.

The evolution of dental implants has a long history that has been briefly described chronologically in the work of Abraham [1]. Dental implants are medical devices that offer a popular and effective way to replace missing teeth. The dental implant system is commonly composed of four parts: implant, abutment, abutment screw, and artificial crown (Figure 1).

Implants are shaped like a screw that is surgically implanted into the jawbone to act as a support for crowns, bridges, or dentures, and to restore the appearance of missing teeth and the person’s ability to chew [3]. The abutment is often connected to the implant with an abutment screw (Figure 1 (right)), and it is used to connect the artificial crown to the implant, which can be fixed mechanically or with special adhesive materials.

**Figure 2:**
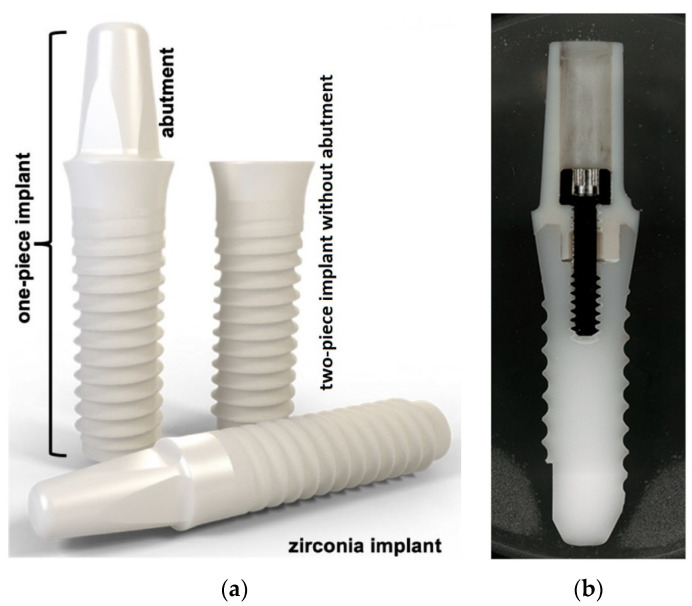
Ceramic implants: a one-piece and two-piece implant with an abutment (**a**) [6], and a two-piece implant with an abutment (**b**). Reproduced from [6,7], with permission from Springer Nature (2023) and MDPI (CC BY-NC 3.0), respectively.

There are two types of dental implants: metal and ceramic. Both have advantages and disadvantages. Metal implants, which have been in use since the 1960s, are usually made of titanium, due to its biocompatibility with the human body. Moreover, titanium implants have been shown to be very safe and effective due to their high strength, light weight, and the durability of titanium. On the other hand, ceramic implants are usually made from zirconium dioxide, which is a “metal free” material that is biocompatible with the human body. Some studies on biomaterials in implantology have shown that zirconium dioxide implants are highly biocompatible, they integrate well with bone and gum tissue, and they have a tendency to reduce plaque buildup [4]. Metallic implant systems contain the parts showed in Figure 1, whereas ceramic implants can be made of one solid piece or two pieces joined with cement or an abutment screw (Figure 2) [5]. Although data show that the difference between the performance of metal- and ceramic-based implants is negligible, many find that they are uncomfortable with utilizing metal-based materials.

**Figure 3:**
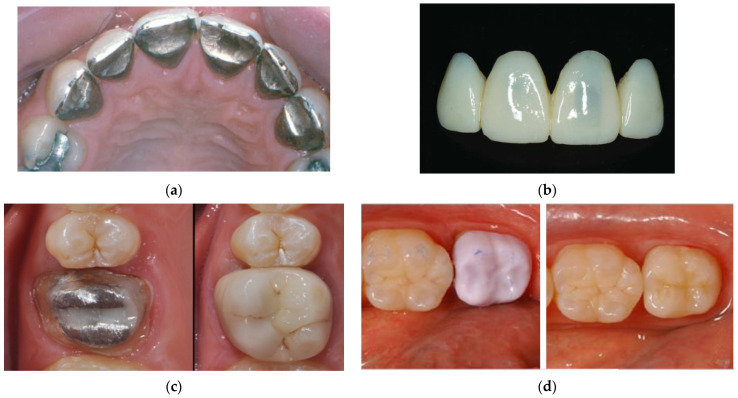
Types of materials for dental restorations: (**a**) metal [8]; (**b**) porcelain [9]; (**c**) ceramic (**right**) [10]; and (**d**) lithium disilicate before (**left**), and after (**right**) crystallization [11]. Reproduced from [8,9,10,11], with permission from Springer Nature (2023), Elsevier (CC BY-NC 4.0), Verizona Publisher (CC BY), and Elsevier (2023), respectively.

The idea behind any type of implant is to merge it with the bone, creating a permanent base for fixed prosthetic restorations. For the latter, four types of materials are used: metal, porcelain, and ceramics based on zirconium dioxide and lithium disilicate (lithium disilicate) (see Figure 3).

Metal restorations (e.g., metal crowns) can be made of silver, gold, titanium, or other metal alloys. Usually, the preferred material is gold, in combination with other metals, such as copper, chromium, or nickel. The choice of gold material for the fabrication of dental restorations is justified by its strength and durability; in fact, gold restorations offer ideal long-term protection for the natural tooth, are easy to remove, and rarely chip [12].

**Figure 4:**
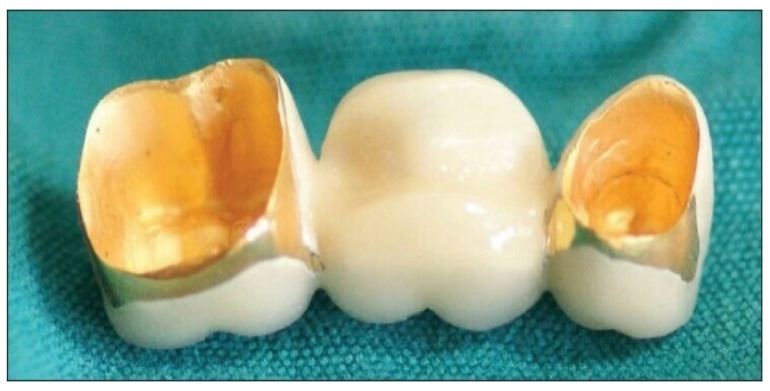
Porcelain fused-to-metal dental restoration. Reproduced from [14] (CC BY-NC-ND 4.0), published by Thieme, 2013.

Porcelain is also among the most popular materials used for dental restorations because it is cheap and provides the best and most natural look. Moreover, porcelain restorations are very easy to clean because there is no metal to trap dirt. Additionally, crowns of this type are not as strong as metal ones, and they can last a long time [13]. Porcelain-fused-to-metal (PFM) restorations, another widely used type of dental restoration, are made of a porcelain outer part fused to a metal inner part, which results in a strong and durable structure (see Figure 4).

Both crowns and bridges can be fabricated as porcelain-fused-to-metal restorations to restore teeth and protect them from further damage or decay. Regarding PFM restorations, the porcelain part offers a desirable natural appearance, while the inner part provides strength and durability due to the mechanical properties of the metal. Although this type of restoration offers a natural appearance, the presence of metal beneath the porcelain can sometimes create a darkish shade or line at the gum line [15]. Additionally, a major disadvantage of this type of restoration is that the porcelain part can chip or crack, and because it is fused to the metal, it can delaminate completely as a result of breakage. However, in this case, the tooth structure suffers no damage, because the metal alloy base underneath remains intact.

With PFM restorations, porcelain can be fused with different types of metals, such as base metal alloys, titanium alloys, or gold alloys. The type of metal used is one of the key factors that determines the final price and quality of PFM restorations. The first group comprises base metals popularly used in dentistry (cobalt, nickel, and chromium), which have a lower price than titanium and gold alloys. Base metals tend to provide a restoration with the minimum level of integrity necessary to satisfy the functional and aesthetic needs of the patient. However, these metals can cause allergic reactions in some people, so dentists must carefully select the type of metal to use in each patient. In the European Union, the dental use of nickel–chromium alloy is currently restricted, and that of the cobalt–chromium alloy will soon be limited because of possible cobalt-related adverse biological effects. To avoid allergic reactions, the dentist may propose the use of titanium alloys or noble-metal alloys (e.g., gold). The properties of titanium alloys are superior to those of base metal alloys, making them prominent materials for implants and other dental prostheses. However, the best results in terms of the appearance, durability, and biocompatibility of PFM restorations are obtained with the use of gold alloys. These are combinations of noble metals (usually a 40% gold and 20% mixture of platinum, silver, and palladium), which have superior properties, and base metals (usually a 40% mixture of copper and tin); such a composition provides great resistance and durability and does not cause allergies. In a gold alloy, the higher the noble metal content, the higher the price, and the better its properties.

In recent decades, ceramic restorations have gained popularity due to the following advantages: high biocompatibility with the human body, absence of allergic reactions due to their metal-free construction, greater comfort than with metal crowns, and natural appearance. Among the ceramic materials used in dentistry, zirconium dioxide initially became the most popular for dental restorations due to its low cost, wear compatibility with natural dentition, and high toughness and strength [16,17,18]. Thus, this material combines hardness and elasticity, which makes it less prone to fracture than PFM restorations, and it has the same desirable appearance as porcelain crowns. However, the strength of sintered zirconium dioxide restorations can cause teeth to wear out easily [19].

A modern material for dental restorations is lithium disilicate, launched in 2005 by Ivoclar Vivadent (Liechtenstein), with the name of IPS™ e.max [20,21]. Its name stands for Esthetic Maximum (i.e., the most aesthetic), and it is a high-quality material [22,23]. IPS™ e.max is a ceramic system based on lithium disilicate glass, which consists of lithium di-oxide, potassium oxide, alumina, quartz, phosphoroxide, and trace elements, and it is characterized by a translucent color and good durability. Restorations made from this material are the most expensive on the market [24]. Moreover, this material is preferred over zirconium dioxide crowns because it is tougher, more durable, and less prone to chipping.

Zirconium dioxide and IPS™ e.max restorations are usually milled from a single block using CAD/CAM technology, allowing the dental restoration to take place during a single visit [24]. CAD/CAM, an acronym for computer-aided design–computer-aided manufacturing (or computer-assisted machining) [25], makes it possible to obtain strong and durable restorations that look exactly like the other teeth, due to the use of digital scanners, which create a three-dimensional model of the oral cavity and teeth [26]. This reduces the number of steps in the process of prosthesis design, creation, and implantation; furthermore, it reduces the manufacturing time, and increases the accuracy and quality of the final product. Moreover, CAD/CAM technology allows for the creation of dental structures with complex shapes and designs, which increases flexibility and accuracy in the treatment of each patient [12]. As a result, the use of this technology increases the design possibilities and production speed for ceramic elements in dentistry.

The purpose of our study was to review research on the materials used for all-ceramic dental restorations, including the ceramic materials used for the production of CAD/CAM blanks, and the related manufacturing processes. This article is divided into six sections, as follows: introduction, materials involved in the manufacturing process of all-ceramic dental restorations, methods for the fabrication of all-ceramic restorations, CAD/CAM technique for the fabrication of dental res-orations, production of ceramic blocks for CAD/CAM dentistry, and conclusions.

## 2. Materials Involved in the Manufacturing Process of All-Ceramic Dental Restorations

The term “all-ceramic” is used when a dental restoration is made entirely of ceramic material. Monolithic, or “uni-layer”, restorations consist of a single ceramic material; bilayered ones, on the other hand, are formed by a ceramic core covered with a ceramic veneer [27,28], where the former supports the restoration, and the latter provides its final shape, shade, and appearance. The major disadvantage of bi-layered restorations lies in the low strength of the bond between the core and the veneer, which makes them prone to delamination and fracture; monolithic restorations are longer-lasting than bi-layered ones because they are composed of only one ceramic material. On the other hand, the aesthetic outcome of bi-layered restorations is superior to that obtained with monolithic ones. Thus, bi-layered ceramic is recommended in cases where a desirable appearance is essential, for example, for anterior teeth, and monolithic ceramic is used when appearance is not as important, such as for posterior teeth.

Over the last 10 years, the most common ceramic materials for producing dental restorations have been zirconium dioxide and lithium disilicate. However, other materials, such as porcelain and alumina, are also used for the production of all-ceramic dental restorations. In this section, we present a short introduction of these materials, along with a brief history of their development.

### 2.1. Porcelain

The idea to use ceramics in dentistry first emerged in 1889, when Charles H. Land patented the all-porcelain “jacket” crown [29], which was designed to protect the entire surface of the broken tooth. However, this type of crown was only introduced in practice during the early 1900s, after E.B. Paulding had significantly improved it, and it was used until the 1950s [30,31]. Although the jacket crown was effective in making the tooth appear whole again, it lacked sturdiness because of microcracking, which happened during the cooling phase of the manufacturing process, and this caused issues for the crown, the underlying tooth structure, and the surrounding gum tissue [30,32]. In order to solve this problem, in 1965, W. McLean and T.H. Hughes [33,34] developed a new type of dental porcelain in which high-strength alumina was added to the felspathic matrix. The obtained reinforced porcelain was twice as strong as the traditional porcelain “jacket” crown; however, its drawbacks were that it could only be used in the anterior region (due to its lower strength) and it presented with a greater opacity [35].

Dental porcelain can be classified into three groups depending on its application (i.e., prosthetic teeth, ceramometallic applications, or all-porcelain restorations [36]), and each porcelain type has a different composition. For instance, dental porcelain for prosthetic teeth, commonly known as high-temperature porcelain, comprises a mixture of quartz, clay, and feldspar powders. On the other hand, feldspathic dental porcelain, which contains powders of potassium feldspar and glass, is used for ceramometallic restorations and fabricating porcelain inlays and veneers. The third type of dental porcelain, known as aluminous porcelain, comprises a mixture, the composition of which is similar to that of feldspathic porcelain, but with aluminum oxide fillers. After sintering, these types of porcelain present similar components, that is, small aluminosilicate crystals (for example, leucite) embedded in silicate glass; thus, they are differentiated by the relative amount of crystals and glass. In porcelain, leucite is an important component due to its influence on hardness, strength, optical properties, and thermal expansion.

### 2.2. Alumina

Alumina is the ceramic material most commonly used in different fields of science and technology due to properties such as wear resistance, strength, and biocompatibility. Alumina, also known as aluminum oxide, has been used as an implant material in the biomedical field for orthopedic and dental reparations since 1964, when Sanhaus first used it for tooth replacements [37]. Since then, alumina has been proposed as a biomaterial in various clinical applications. Early clinical applications showed a fracture rate as high as 13% [38]. In this study, it was found that material failure was due to the fact that ceramic restorations could not be sintered to full final density. Only during the late 1980s was a new generation of alumina developed; the higher density and smaller grains resulted in a reduced fracturing rate, of less than 5% [39]. The third generation of alumina appeared in 1995, with the introduction of hot isostatic pressing technology to the manufacturing process, which provided ceramic restorations with a finer microstructure, fuller density, higher purity, and superior mechanical properties [40,41]. However, even though alumina has a very high resilience to fracturing, fractures are still frequent in clinical use [42,43]. Since the introduction of third-generation alumina, there have been significant advances in the mechanical properties and manufacturing methods of alumina-based ceramic materials [44,45].

### 2.3. Zirconium Dioxide

Zirconium dioxide, also known as zirconium dioxide (ZrO_2_), is a ceramic material which became popular for dental restorative treatments due to its properties of excellent biocompatibility, appearance and wear properties, superior toughness, fatigue resistance, and strength [46].

**Figure 5:**
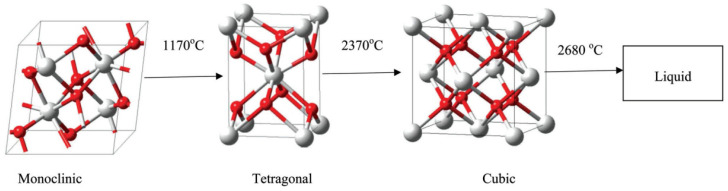
Zirconium dioxide phase transformation with variations in temperature. Reproduced from [48], with permission from Springer Nature, 2023.

At room temperature, zirconium dioxide is in a monoclinic (m) phase, which, upon heating, is stable up to 1170 °C (see Figure 5). From this temperature to 1206 °C, the trans-formation from the m phase to tetragonal (t) phase occurs; the t phase remains stable up to approximately 2370 °C. Subsequent heating leads to the transformation from the t phase to the cubic (f) phase, which is stable until the melting point of zirconium dioxide is reached [47].

When heated zirconium dioxide is cooled from 1052 °C to 950 °C, the transformation from the tetragonal phase to monoclinic phase, known as martensitic transformation [49], takes place; it is characterized by a relatively large increase in the volume of the monoclinic phase, approximately 4% compared with that of the tetragonal phase, which leads to monolithic destruction due to the very high internal stress that arises. In order to prevent this transformation, zirconium dioxide is often modified with various oxides, such as ceria (CeO_2_), alumina (Al_2_O_3_), magnesia (MgO), calcia (CaO), and yttria (Y_2_O_3_). The latter is the most commonly used, since yttria successfully stabilizes the transformation of the crystal structure by preventing the formation of the monoclinic phase during cooling, and it maintains the tetragonal and cubic phases; this prevents the formation of cracks, improving the physical properties of zirconium dioxide. Thus, zirconium dioxide modified with yttria is widely used in biomedical purposes. It is necessary to take into account that the effectiveness of zirconium dioxide stabilization depends on the amount of stabilizer used; for instance, cubic phase ZrO_2_ requires a higher amount of stabilizer than tetragonal phase ZrO_2_. 

**Figure 6:**
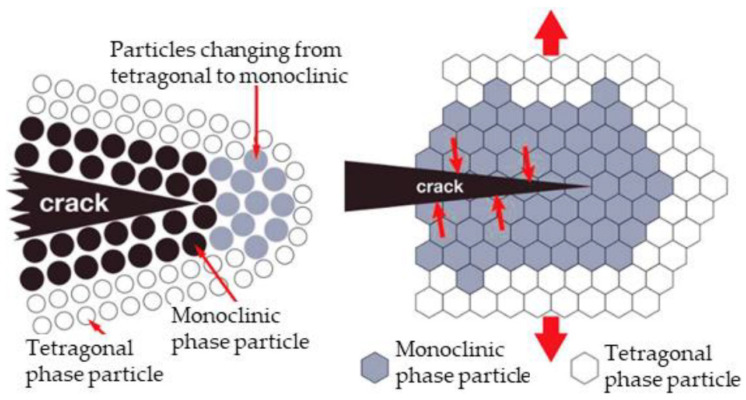
Transformation toughening mechanism in zirconium dioxide. Reproduced from [53] (CC BY-NC 3.0), published by MDPI, 2020.

Another method to stabilize tetragonal phase ZrO_2_ involves creating a surface energy difference by decreasing the crystal size to a value < 0.3 μm [50]. However, the zirconium dioxide used for the production of dental material is metastable (at room temperature), tetragonal, partially stabilized zirconium dioxide (PSZ). The term metastable refers to the fact that in the material, there is accumulated energy that can be used in the transformation to the monoclinic phase ZrO_2_. For example, the accumulation of extremely high stress during crack propagation is sufficient for zirconium dioxide grains in the vicinity of the crack tip to transform into the monoclinic phase. This produces a local increase, at a volume of 4%, which compresses the crack and prevents its propagation, thus increasing the toughness of the material (see Figure 6). This mechanism is known as transformation toughening [49]. After sintering, the average biaxial flexural strength of yttria-stabilized zirconium dioxide lies in the range of 850 MPa to more than 1000 MPa [51,52].

In dental applications, dental restorations can be obtained from monolithic zirconium dioxide, high-translucency zirconium dioxide, or porcelain-layered zirconium dioxide. Monolithic zirconium dioxide restorations, also known as solid, full-contour zirconium dioxide restorations, are commonly used for areas that are less aesthetically important, such as for the posterior teeth, due to their tendency to be opaque, monochromatic, and the fact that they have a lack of translucency and fluorescence [54,55]. They are excellent for masking discolored dental preparations, for example, in those that have darkened due to chronic diseases or previous dental treatments. High-translucency zirconium dioxide restorations are more aesthetically pleasing than the monolithic type due to their natural and vibrant translucency and opalescent characteristics, which make them suitable for anterior teeth. Despite high-translucency zirconium dioxide being weaker than its monolithic counterpart, the biaxial flexural strength of the former is still high and ranges from 590 MPa to 720 MPa and above. Porcelain-layered zirconium dioxide restorations are designed to have the appearance of porcelain and the structural strength of zirconium dioxide, and thus, they are fabricated using a porcelain veneer over the ceramic coping. The aesthetic quality of these types of restorations is similar to that observed in high-translucency zirconium dioxide. However, the weak point of these restorations is located precisely where the porcelain layer is used, since this material has a bending resistance value of over 100 MPa.

### 2.4. Lithium Disilicate

Lithium disilicate, as a ceramic for dental applications, was discovered in 1988 by Ivoclar Vivadent (Liechtenstein), with the name of IPS™ Empress 2 [56]. This material was a type of particle-filled glass, commonly known as glass–ceramic, and it contained around 70% crystalline lithium disilicate filler [57,58]. IPS™ Empress 2 showed a more uniform crystalline distribution, and it had fewer defects when it was used in conjunction with a pressure casting procedure [59]. In 2005, the production process of Empress 2 was optimized, allowing for the emergence of a new ceramic material, IPS™ e.max Press, which led to the discontinuation of IPS™ Empress 2 in 2009 [60]. Lithium disilicate ceramics have made a revolutionary contribution to the field of all-ceramic restorations due to the improvements made to the aesthetic and mechanical properties of glass–ceramics [61]. In lithium disilicate, the lithium oxide crystals (Li_2_O) are dispersed in a glassy matrix of silica (SiO_2_), providing flexural strength values of up to 440 MPa and inhibiting the propagation of cracks due to their interlocking orientation [62]. Due to their excellent aesthetic properties, restorations based on lithium disilicate are used to repair anterior teeth [63]. However, it has been established that the failure rate of these restorations can reach 3.3%, as they can show damage such as ceramic fracturing, chipping [64], and rupturing of the core ceramic [65].

In 2006, the company Ivoclar Vivadent (Liechtenstein) released lithium disilicate glass–ceramic IPS™ e.max CAD, which was specifically designed for CAD/CAM production [66]. This material is purchased in a “blue state”, and it is primarily composed of meta-silicates (Li_2_SiO_3_; ~40%), in addition to lithium disilicate crystal nuclei (Li_2_Si_2_O_5_) [67] (see Figure 3d (left)).

IPS™ e.max CAD is milled in its partially crystallized “blue state”, which is characterized by a moderate flexural strength (~130 MPa), fracture toughness (0.9–1.25 MPa·m^1/2^), and Vickers hardness (5400 MPa), resulting in higher cutting efficiency, easier and faster workability, and lower wear of the milling tools [22,59]. Once the milling process is completed, the restoration obtained is heat-treated at 840–850 °C, for 20–25 min, under a vacuum, to transform the metasilicate crystals into ~70% lithium disilicate and to obtain a fully crystallized microstructure; this increases the flexural strength up to values of ~260 MPa and the fracture toughness up to values of 2.5 MPa·m^1/2^.

It was reported that the manufacturing process of IPS™ e.max CAD and IPS™ e.max Press did not seem to affect their mechanical properties; for instance, their flexural strengths resulted to be similar. However, it was observed that translucency significantly influenced the flexural strength of CAD-processed materials only [68].

## 3. Methods for the Fabrication of All-Ceramic Restorations

In dentistry, different methods are used to manufacture all-ceramic restorations. These methods include the conventional technique, the hot-pressing technique, the dry-pressing method, the slip-casting and glass infiltration method, and CAD/CAM.

### 3.1. Conventional Technique

**Figure 7:**
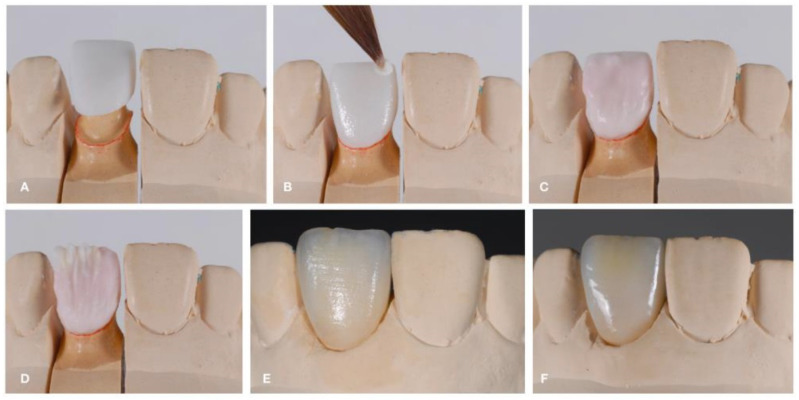
Conventional technique. (**A**) Polycrystalline Framework; (**B**) Feldspathic ceramic build-up (wash bake); (**C**) Feldspathic ceramic build-up (intensive chrome and dentin); (**D**) Feldspathic ceramic build-up (enamel layer); (**E**) Feldspathic ceramic after firing; (**F**) Feldspathic ceramic after a thermal and mechanical glaze. Reproduced from [70] (CC BY-NC 3.0), published by MDPI, 2022.

The conventional technique is also known as the layering technique. This technique is based on the successive stacking and sintering of ceramic materials. Sintering is the process of firing the stacked ceramic material at a high temperature to ensure optimal densification, which occurs through pore elimination and viscous flow when the firing temperature is reached. During this method, the ceramic material, initially in powder form, is mixed with water or a water–glycerin mixture to form a slurry (a malleable mass). Then, the slurry is applied in layers to a dye material (a platinum foil, a refractory dye, or a metal) [69]. The excess liquid in the applied layers is brought to the surface with a vibrating motion, and then eliminated using an absorbent tissue to form a “green state”. The as-obtained green body can be carved and shaped to form the contours of the core or restoration. Once the restoration green body is obtained, it is sintered at high temperatures to obtain a hard material with greater mechanical and optical characteristics. Ceramco 3^®^ by Dentsply and VM® 13 by Vita are materials fabricated using this method. The conventional technique is the most common fabrication method for the veneer ceramic in ceramometallic restorations. Figure 7 shows the steps of the conventional technique.

### 3.2. Heat Hot-Pressing Technique

**Figure 8:**
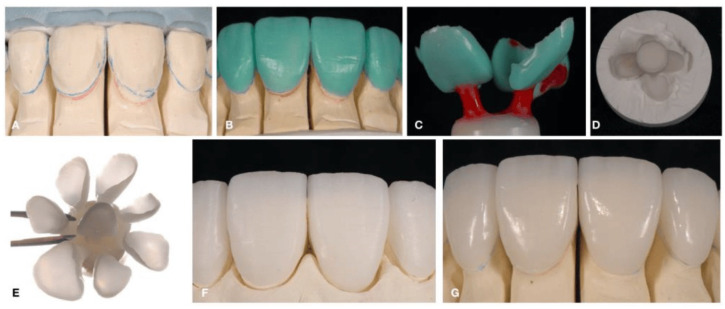
Hot-pressing technique. (**A**) Stone model and putty matrix from diagnostic wax-up; (**B**) Wax-up; (**C**) Sprueing, investing, and pressing; (**D**) Divesting; (**E**) Removing the reacting layer; (**F**) Staining, firing, and glaze; (**G**) Final restoration. Reproduced from [70] (CC BY-NC 3.0), published by MDPI, 2022.

The hot-pressing technique creates products that are also known as pressable ceramics, and it involves heating the ceramic material at a high temperature and applying external pressure in order to sinter it and provide the final shape for the restoration. In particular, to fabricate ceramic restorations, this method involves lost-wax casting. First, a wax model of the restoration to be fabricated is prepared, and then, it is covered with refractory dye materials [71]. A mold is obtained by eliminating the wax. Then, a ceramic block (ingot) is heated at a high temperature, near the softening point of the ceramic, and pressed into the mold using a refractory plunger, filling the mold with softened ceramic. The as-fabricated body presents a good dispersion of the crystalline phase within the glassy matrix, and it can be either used as a substrate for conventional feldspathic porcelain build-up or made into full-contour crowns. The advantage of this method is that it can be employed by dental technicians following the fabrication steps mentioned above (i.e., investing, wax elimination, and casting). Materials that can be used for this technique are leucite (IPS Empress; Ivoclar, Liechtenstein), lithium disilicate (IPS Eris; Ivoclar, Liechtenstein), spinel (Alceram; Innotek Dental Corp, Lakewood, CA, USA) [72], and ceramics based on them [73] (for instance, IPS™ Empress ceramics). The main disadvantage of this method is the high cost of the equipment, due to the necessity to use a specially automated pressing furnace. Figure 8 shows the steps of the hot-pressing technique.

### 3.3. Dry-Pressing Method

This is one of the methods which enables polycrystalline (alumina or zirconium dioxide) restorations to be fabricated. First, a life-sized natural model of the restoration to be created is obtained. Then, this model is scanned to obtain an enlarged (oversized) 3D model in order to fabricate a mold with oversized dimensions. Afterwards, ceramic powder is pressed into the mold, obtaining a green body, which is sintered. During sintering, the green body shrinks to the required dimensions. The dye model is oversized by around 12–20% to compensate for the shrinkage that occurs during sintering. Examples of ceramic materials produced with this method are Procera^®^ Alumina and Procera^®^ Zirconia by Vita [74].

### 3.4. Slip-Casting and the Glass Infiltration Method

**Figure 9:**
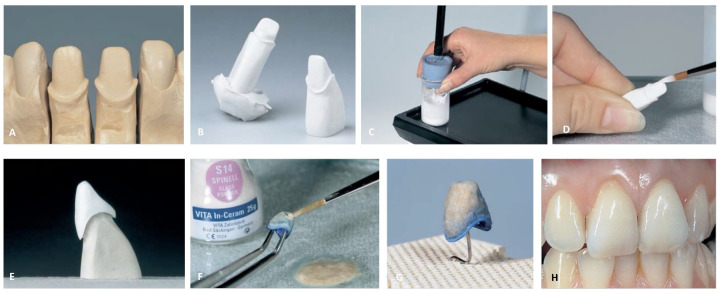
Slip-casting and glass infiltration. (**A**) Split model from high strength class of stone; (**B**) Refractory models; (**C**) Slip preparation; (**D**) Slip application; (**E**) Sintered framework on refractory model; (**F**) Glass application; (**G**) Glass infiltration firing; (**H**) Final restoration. Adapted from and open access material [76], published by Vita, 2005.

During the slip-casting and glass infiltration method, ceramic restorations are fabricated using the following components: ceramic powder (such as alumina (In-Ceram^®^ Alumina; Vident, Baldwin Park, CA, USA), spinel-toughened alumina (In-Ceram^®^ Spinell; Vident, Baldwin Park, CA) or zirconium dioxide-toughened alumina (12Ce-TZP-Al2O3; In-Ceram^®^ Zirconia; Vident Baldwin Park, CA), yttria-stabilized tetragonal zirconium dioxide polycrystalline (3Y-TZP; Cercon^®^; Dentsply, Charlotte, NC, USA)), and glass [72]. Firstly, an aqueous slurry (slip) of small ceramic crystals is condensed with a refractory dye. Due to the pores present in the latter, the water from the ceramic slip is easily absorbed through capillary action. The thickness is gradually increased until the restoration is completely formed; then, the latter is shaped and finally sintered at a high temperature using the refractory dye. After sintering, the restoration can be easily separated from the refractory dye as the latter shrinks away from the condensed slip. Then, the porous core of the sintered restoration is glass-infiltrated, that is, the glass material is melted and drawn into the pores at a high temperature through capillary action. As a result, a microstructure that consists of a crystalline infrastructure filled with glass is obtained [75]. The main advantage of slip-cast ceramics is their high strength; disadvantages include high opacity (with the exception of spinel-based materials) and long processing times. Figure 9 shows the steps of slip-casting and glass infiltration.

### 3.5. CAD/CAM

CAD/CAM dentistry is a field of dentistry and prosthodontics that uses computer-aided design (CAD) and computer-aided manufacturing (CAM) technologies to improve the design and creation of dental restorations, such as inlays, onlays, veneers, and crowns [25]. CAD/CAM methods, now an important part of dentistry, are techniques in which the design and fabrication of ceramic restorations are carried out using computer software [77]. When the tooth is prepared, it is optically scanned, and its image is computerized. From this image, the restoration is designed with a special computer program, and then machined in a computer numerical control (CNC) milling machine. The advantages of these methods include a reduction in clinical time and cross-infections between the clinic and the laboratory, and a reduction in, or elimination of, patient discomfort, particularly when intraoral scanning (digital impression) is used. Using CAD/CAM systems, the doctor and/or technician can examine the restoration before the beginning of the milling process to verify its accuracy. Moreover, this technology allows dental restorations to be manufactured individually at the dentist’s office, or centrally in a dental laboratory. Disadvantages of CAD/CAM systems include the amount of investment needed, and the cost of equipment and its maintenance, which makes this manufacturing technique more expensive than others.

Tetrasilicic fluoromica (DICOR MGC; Dentsply International, Inc., York, PA, USA), Sanidine (Vitablocs^®^; Mark II Vident, Baldwin Park, CA, USA), Sanidine (Vita-Celay; Vi-dent, Baldwin Park, CA, USA), Alumina (In-Ceram^®^ AL; Vident, Vident, Baldwin Park, CA), Alumina (Procera All Ceram; Nobel Biocare, NY, USA), Leucite (IPS Empress^®^ CAD; Ivoclar, Liechtenstein), Lithium disilicate (IPS e.max CAD; Ivoclar, Liechtenstein), and Y-TZP (Lava CAD/CAM; 3M ESPE, St. Paul, MN, USA) are examples of ceramic materials used in this method.

**Figure 10:**
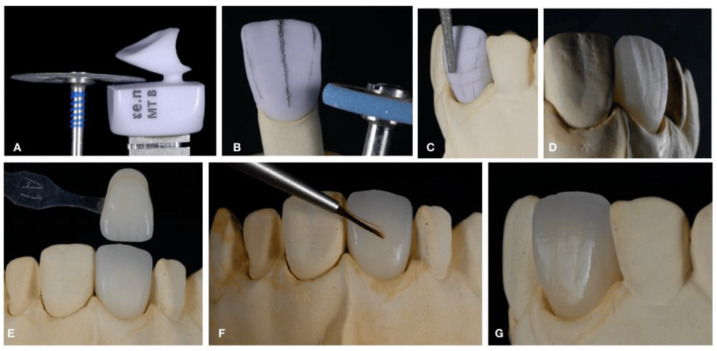
Milled technique. (**A**) Cutting out a milled restoration from a CAD/CAM block; (**B**) Controlling the margin’s thickness (emergence profile); (**C**) Controlling macro- and micro-textures (finishing); (**D**) After crystallization; (**E**) Stain technique; (**F**) Glaze; (**G**) Final restoration. Reproduced from [70] (CC BY-NC 3.0), published by MDPI, 2022.

Dental blocks are machined during a milling process, in which burrs and milling tools are used [78]. Different coatings are used to reduce tool wear when machining all-ceramic restorations [79], which has a number of advantages. For example, it saves time, as the use of a coating allows for an increase in processing speed; it saves money, as the coatings used maximize tool life; and finally, the use of coated tools ensures that the machined surface is of a high quality [80,81,82]. Figure 10 shows the steps of the milling technique.

## 4. CAD/CAM Technique for Fabrication of Dental Restoration

The use of CAD/CAM in dentistry started in 1985, when the first CAD/CAM inlay was fabricated from a feldspathic ceramic block [83]. Among the commonly used methods for the fabrication of all-ceramic restorations, the CAD/CAM technique has been the most popular one for the past 10 years. Its popularity is related to its advantages over other techniques, namely, ease of use, speed, and quality.

Ease of use: CAD/CAM systems are designed and created to be easily used and adapted to any task. Additionally, most CAD/CAM systems use software that is easy to use and guides the operator through the milling process. Moreover, most manufacturers of CAD/CAM systems create restoration variants that generally require few or no modifications. Another benefit of using CAD/CAM systems is that the scans of teeth and the designs of the restorations are digitally stored on the computer, whereas standard stone models take up a great deal of space and can deteriorate if stored improperly.

Speed: With CAD/CAM, the restoration can be produced in a single visit, whereas with traditional technologies, two or three weeks are required to prepare the impression. This is possible when clinics have their own scanner and milling machine, as they can begin the restoration manufacturing process immediately. This means that provisional restorations, which also require time to manufacture and adjust, are no longer necessary. Digital scans of patient’s teeth are much easier and faster to obtain than conventional prints, since the manufacturing process of the latter requires different production stages (casting, waxing up, investing, casting, and firing), which are not necessary when scanning is used. For instance, half-arch impressions can be obtained in 40 s, whereas full-arch impressions can take up to 2 min [84]. Similarly, the design and fabrication processes are faster; for instance, a full-contour crown can be fabricated in 6 min using the milling method.

Quality: The restorations obtained with CAD/CAM are of extremely high quality due to the measurements obtained during scanning, and their manufacturing precision is very high. Furthermore, the appearance of CAD/CAM restorations is very natural, due to the fact that the ceramic blocks used are available in a very wide range of shades, and their translucency is very similar to natural enamel. In addition, ceramic restorations result in less wear on opposing teeth since they are less abrasive than conventional and hybrid composite resins. In addition to the above, the ceramic blocks used in CAD/CAM dentistry are free of internal defects, which makes them more durable, thus allowing for the production of more durable restorations for dental patients.

Despite the abovementioned significant advantages of the CAD/CAM technique, there are also several disadvantages. First, the initial cost of the CAD/CAM system (equipment and software) is high; furthermore, the operator of this system needs to invest considerable time and money on training. This means that clinics or laboratories that have few orders for manufacturing restorations likely face difficulties in amortizing their investments. Second, digital scanning requires the same type of soft-tissue management, retraction, moisture control, and hemostasis that is pivotal for conventional impressions. Finally, current digital impression systems do not save much time due to the presence of multiple steps. In fact, clinics or laboratories that use certain types of scanners first send the images to an external facility for a cleanup process, after which, a dental technician performs margin setting. Next, the images are returned to the clinician’s dental laboratory for review before being sent back to the facility for model milling. Finally, the models and dyes are then sent to the clinician’s dental laboratory for the fabrication of the restoration.

The CAD/CAM technique involves three processing steps. First, an impression of the prepared tooth and the surrounding tissue is taken either digitally or with a conventional method. If the latter is used, a stone replica is usually obtained and then scanned to develop a digital impression. Second, the digital impression is processed by a computer in order to design the 3D model. Third, the processed information is used to control and guide a milling machine that is connected to a computer, thereby producing the planned restoration [85]. With some techniques, digital impressions can also be used to scan the prepared tooth or teeth and the occlusion of the opposing jaw, so that an interocclusal record is not required.

The machining process in CAD/CAM systems can be classified into two types: soft and hard machining. This classification depends on the type of ceramic block to be ma-chined, which can be partially or fully sintered. Soft machining is also known as partially sintered-state milling, since the ceramic blocks contain partially sintered polycrystalline ceramics. Enlarged cores or restorations are obtained by milling these blocks, where the dimensions are then reduced as necessary during the subsequent sintering process at high temperatures; this occurs to achieve a fully sintered state. Examples of soft-machined ceramics are the Lava^®^ CAD/CAM system and IPS e.max ZirCAD^®^ (Ivoclar Vivadent, Liechtenstein).

Hard machining is also known as fully sintered-state milling, since the machined ceramic blocks are in a fully sintered state and do not require additional heat treatment. Restorations produced using this method have a superior fit [86]. The main disadvantages of this method include the wearing down of the cutting tool and a lengthy fabrication process. Fully sintered zirconium dioxide dental blocks are machined by using a grinding process in which grinding burrs and/or grinding tools are used. However, the use of fully sintered dental blocks is almost always avoided because of the difficulty and costs associated with the milling process.

Laser milling processing is another machining method for obtaining fully sintered dental blocks in which laser evaporative heating is used to give the necessary shape and dimension to dental restorations.

During dental restoration production, in addition to the presented methods for machining ceramic materials, the concept of using additive technologies is becoming increasingly popular [87]. Every year, the number of publications on the use of 3D printing in dental laboratories for the production of dentures increases [88]. With new developments in the additive manufacturing of ceramic parts, it is likely that innovative approaches to the production of natural-looking dental restorations will appear in the very near future.

## 5. Production of Ceramics Materials for CAD/CAM Dentistry

As mentioned above, the most used materials in CAD/CAM dentistry are ceramics based on zirconium dioxide and lithium disilicate (glass–ceramics). In this section, we briefly detail the stages of the production of these materials.

### 5.1. Production of Zirconium Dioxide CAD/CAM Blocks

The production of zirconium dioxide CAD/CAM blocks is based on the dry-pressing process, a simple method that has the advantage of offering easy batch production, in addition to the fact that the ceramic blocks obtained have few defects and are of good quality, which is convenient for mechanized and automatic production. This production process is generally composed of the following steps: raw material process, green-forming process, pre-sintering, block-forming process, and inspection and packaging. These steps are briefly detailed below [89].

#### 5.1.1. Raw Material Process

During this step, fine-grained powder is obtained from the initial zirconium oxide raw powder. Two methods can be used to achieve this (i.e., wet and dry processes). During the wet process, a liquid medium is used to grind the initial powder into a fine-grained product and prepare a suspension or zirconium paste, which can be used to realize the zirconium granulation process. During the dry process, grinding bodies are employed to obtain fine grains with a size of around 40 nm.

#### 5.1.2. Green-Forming Process

**Figure 11:**
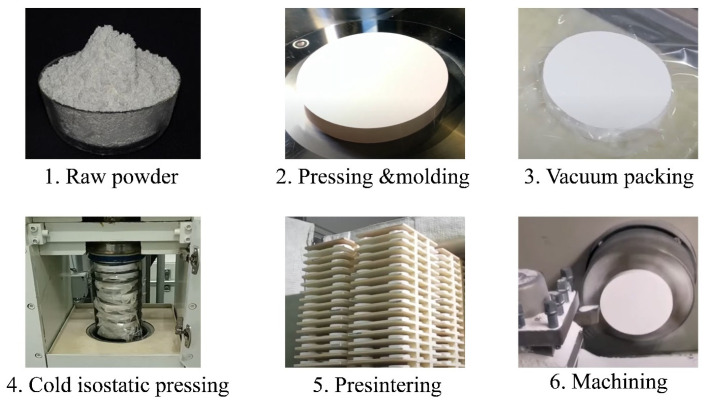
Steps for the production of ceramic blocks for the CAD/CAM dentistry.

The prepared mixture of dry fine-grained powder is isostatically pressed to obtain the initial shape of the blank through consolidation. During this step, the particles are forced to unite homogeneously; thus, porosity is reduced. The green-forming process includes two stages: uniaxial pressing and isostatic pressing (see Figure 11). The former is applied to obtain the preliminary size and shape of the ceramic blank. It is carried out in a mechanical press with a metal mold. After uniaxial pressing, the formed preliminary ceramic blank (green body) is removed from the mold and placed in a vacuum bag. The term ‘green body’ refers to the fact that the obtained blank is in a non-sintered state. Then, during the second stage of the forming process, the vacuum-packed blank is isostatically pressed to obtain a density of 90–95%. There are two main procedures for isostatic pressing: cold and hot isostatic pressing, abbreviated CIP and HIP, respectively. Cold isostatic pressing is the most used procedure for the production of most zirconium blocks. Although CIP requires a successive pre-sintering step, with hot isostatic pressing, the high temperature and pressure are simultaneously applied, so the zirconium dioxide block obtained with HIP is already fully sintered.

#### 5.1.3. Pre-Sintering

When employing CIP, the obtained ceramic green body cannot yet be used for dental zirconium dioxide CAD/CAM blocks, as a pre-sintering stage is necessary to achieve the minimum mechanical properties of the ceramic blank, which prevents its destruction during transport, handling, or machining. After the pre-sintering process, the ceramic blank is partially sintered. The ceramic blocks obtained with HIP do not need to be subjected to a pre-sintering process, since they are already fully sintered.

#### 5.1.4. Block Forming Process

The final dimensions and shapes of the dental blocks are commonly obtained by using subtractive manufacturing. In subtractive manufacturing, the unwanted part of block material is removed using a cutting process, thus obtaining CAD/CAM blocks. For in-stance, partially sintered zirconium dioxide dental blocks are machined by using a milling process, in which burrs and milling tools are used. Depending on the required solution and indications, dental CAD/CAM blocks can be produced in different forms, such as disc-shaped, cylindrical, boxed, dental-arch-shaped (“horseshoe” type of Amann–Girrbach dental CAD/CAM systems), and tooth-morphology pre-shaped blocks.

#### 5.1.5. Inspection and Packing

Finally, metrological control, visual inspection, and proper packaging are the processes of the final stage of the production of zirconium dioxide CAD/CAM blocks. It should be noted that in the case of fully sintered zirconium dioxide blocks, these checks are carried out after the HIP process.

### 5.2. Production of Glass–Ceramic CAD/CAM Blocks

The industrial production of glass–ceramic CAD/CAM blocks comprises almost the same steps as that of zirconium dioxide CAD/CAM blocks. The company, Ivoclar Vivadent, published a video in which the production of lithium disilicate CAD/CAM blocks is shown [90].

The production of glass–ceramic CAD/CAM blocks is generally composed of the fol-lowing steps: raw material process, green-forming process, heating process, inspection, and packaging. These steps are briefly detailed below.

#### 5.2.1. Raw Material Process

The raw material process for glass–ceramics is more complex than that of zirconium dioxide CAD/CAM blocks. During this process, the first stage includes the preparation of the basic powder mixture, in which different compounds are mixed in order to obtain the desired color composition. The obtained basic powder mixture is melted into glass at a temperature of around 1500 °C, which is then immediately quenched in a cold water bath. Afterwards, the cooled glass is finely ground and then sintered in a furnace in order to transform it into glass–ceramic. The last operation in the raw material process consists of finely grinding the glass–ceramic with ball mills. It is worth noting that at this stage, quality control is crucial to further processing, and it includes determining the composition of the resulting powder and assessing whether the obtained mechanical and optical properties perfectly match the desired properties.

#### 5.2.2. Green-Forming Process

The green-forming process of glass–ceramic is the same as that of zirconium dioxide CAD/CAM blocks. The prepared mixture of dry fine-grained powder is uniaxially pressed into a metal mold to obtain the initial box shape of the blank. As in the case of zirconium dioxide powder, during consolidation, the particles are forced to unite homogeneously; thus, porosity is reduced. When the blank (i.e., the green body) is obtained, its dimensions and shape are checked.

#### 5.2.3. Heating Process

For IPS Empress blocks, the blanks prepared from powder are fired in a vacuum furnace at 1000 °C. On the other hand, for IPS e.max, the blanks prepared from powder are partially ceramized with a complex procedure, as a result of which, IPS e.max blocks obtain their characteristic blue color.

## 6. Conclusions

The materials used in dentistry for the fabrication of all-ceramic restorations have undergone significant and rapid developments over the last two decades. Among the most common ceramic materials in dentistry are those based on zirconium and lithium disilicate. Alongside the advances in ceramic materials for dental restorations, technologies for processing such materials (e.g., CAD/CAM systems) have also undergone rapid and efficient developments.

Due to the optical properties of lithium disilicate, it is considered the best material in terms of appearance for dental restorations. On the other hand, zirconium-based materials offer high mechanical properties, and they are frequently used to manufacture all-ceramic restorations, which do not have high aesthetic requirements.

Since both zirconium-based and lithium disilicate materials are in great demand, they are commonly machined with CAD/CAM systems, which offer the possibility of manufacturing all-ceramic dental restorations in a very short period of time; the resulting products have high mechanical properties and an unmatched natural-looking appearance.

As noted in our review, for the fabrication of all-ceramic dental restorations with excellent properties, it is necessary to use ceramic blocks obtained using specific techno-logical processes, which, despite their apparent simplicity, require accurate control at each stage, especially during the preparation of raw ceramic powder. These manufacturing methods require highly homogeneous initial materials to produce high-density final restorations.

It is expected that, in the future, in addition to the further development of existing ceramic materials, which aims to improve their capabilities and mitigate the impact of their disadvantages, current technological processes for the production of ceramic dental restorations will also be perfected; in addition, new manufacturing methods, including techniques using additive technologies, are expected to appear.

## Data Availability

Not applicable.
